# Immersion in water in labour and birth

**DOI:** 10.1590/1516-3180.20131315T2

**Published:** 2013-10-01

**Authors:** Elizabeth R. Cluett, Ethel Burns

## Abstract

**BACKGROUND:**

Enthusiasts suggest that labouring in water and waterbirth increase maternal relaxation, reduce analgesia requirements and promote a midwifery model of care. Critics cite the risk of neonatal water inhalation and maternal/neonatal infection.

**OBJECTIVES:**

To assess the evidence from randomised controlled trials about immersion in water during labour and waterbirth on maternal, fetal, neonatal and caregiver outcomes.

**METHODS:**

*Search methods:* We searched the Cochrane Pregnancy and Childbirth Group's Trials Register (30 June 2011) and reference lists of retrieved studies.

*Selection criteria:* Randomised controlled trials comparing immersion in any bath tub/pool with no immersion, or other non-pharmacological forms of pain management during labour and/or birth, in women during labour who were considered to be at low risk of complications, as defined by the researchers.

*Data collection and analysis:* We assessed trial eligibility and quality and extracted data independently. One review author entered data and the other checked for accuracy.

**MAIN RESULTS:**

This review includes 12 trials (3,243 women): 8 related to just the first stage of labour: one to early versus late immersion in the first stage of labour; two to the first and second stages; and another to the second stage only. We identified no trials evaluating different baths/pools, or the management of third stage of labour. Results for the first stage of labour showed there was a significant reduction in the epidural/spinal/paracervical analgesia/anaesthesia rate amongst women allocated to water immersion compared to controls (478/1,254 versus 529/1,245; risk ratio (RR) 0.90; 95% confidence interval (CI) 0.82 to 0.99, six trials). There was also a reduction in duration of the first stage of labour (mean difference -32.4 minutes; 95% CI -58.7 to -6.13). There was no difference in assisted vaginal deliveries (RR 0.86; 95% CI 0.71 to 1.05, seven trials), caesarean sections (RR 1.21; 95% CI 0.87 to 1.68, 8 trials), use of oxytocin infusion (RR 0.64; 95% CI 0.32 to 1.28, 5 trials), perineal trauma or maternal infection. There were no differences for Apgar score less than 7 at 5 minutes (RR 1.58; 95% CI 0.63 to 3.93, 5 trials), neonatal unit admissions (RR 1.06; 95% CI 0.71 to 1.57, three trials), or neonatal infection rates (RR 2.00; 95% CI 0.50 to 7.94, five trials). Of the 3 trials that compared water immersion during the second stage with no immersion, one trial showed a significantly higher level of satisfaction with the birth experience (RR 0.24; 95% CI 0.07 to 0.80). A lack of data for some comparisons prevented robust conclusions. Further research is needed.

**AUTHORS' CONCLUSIONS:**

Evidence suggests that water immersion during the first stage of labour reduces the use of epidural/spinal analgesia and duration of the first stage of labour. There is limited information for other outcomes related to water use during the first and second stages of labour, due to intervention and outcome variability. There is no evidence of increased adverse effects to the fetus/neonate or woman from labouring in water or waterbirth. However, the studies are very variable and considerable heterogeneity was detected for some outcomes. Further research is needed.

**This is the abstract of a Cochrane Review published in the Cochrane Database of Systematic Reviews (CDSR) 2009, issue 2, Art. No.CD000111. DOI:** 10.1002/14651858.CD000111.pub3 (http://cochrane.bvsalud.org/cochrane/main.php?lib=COC&searchExp=Immersion%20and%20in%20and%20water%20and%20in%20and%20labour%20and%20birth&lang=pt). For full citation and authors details, see reference 1.

The abstract (English, French, Chinese and Portuguese languages) is available from: http://onlinelibrary.wiley.com/doi/10.1002/14651858.CD000111.pub3/abstract
